# Ultrasound Microbubble Treatment Enhances Clathrin-Mediated Endocytosis and Fluid-Phase Uptake through Distinct Mechanisms

**DOI:** 10.1371/journal.pone.0156754

**Published:** 2016-06-08

**Authors:** Farnaz Fekri, Ralph Christian Delos Santos, Raffi Karshafian, Costin N. Antonescu

**Affiliations:** 1 Department of Chemistry and Biology, Ryerson University, Toronto, Ontario, Canada; 2 Department of Medical Physics, Ryerson University, Toronto, Ontario, Canada; 3 Graduate Program in Molecular Science, Ryerson University, Toronto, Ontario, Canada; 4 Institute for Biomedical Engineering, Science and Technology (iBEST), a partnership between Ryerson University and St. Michael’s Hospital, Toronto, Ontario, Canada; 5 Keenan Research Centre for Biomedical Science of St. Michael’s Hospital, Toronto, Ontario, Canada; Institut Curie, FRANCE

## Abstract

Drug delivery to tumors is limited by several factors, including drug permeability of the target cell plasma membrane. Ultrasound in combination with microbubbles (USMB) is a promising strategy to overcome these limitations. USMB treatment elicits enhanced cellular uptake of materials such as drugs, in part as a result of sheer stress and formation of transient membrane pores. Pores formed upon USMB treatment are rapidly resealed, suggesting that other processes such as enhanced endocytosis may contribute to the enhanced material uptake by cells upon USMB treatment. How USMB regulates endocytic processes remains incompletely understood. Cells constitutively utilize several distinct mechanisms of endocytosis, including clathrin-mediated endocytosis (CME) for the internalization of receptor-bound macromolecules such as Transferrin Receptor (TfR), and distinct mechanism(s) that mediate the majority of fluid-phase endocytosis. Tracking the abundance of TfR on the cell surface and the internalization of its ligand transferrin revealed that USMB acutely enhances the rate of CME. Total internal reflection fluorescence microscopy experiments revealed that USMB treatment altered the assembly of clathrin-coated pits, the basic structural units of CME. In addition, the rate of fluid-phase endocytosis was enhanced, but with delayed onset upon USMB treatment relative to the enhancement of CME, suggesting that the two processes are distinctly regulated by USMB. Indeed, vacuolin-1 or desipramine treatment prevented the enhancement of CME but not of fluid phase endocytosis upon USMB, suggesting that lysosome exocytosis and acid sphingomyelinase, respectively, are required for the regulation of CME but not fluid phase endocytosis upon USMB treatment. These results indicate that USMB enhances both CME and fluid phase endocytosis through distinct signaling mechanisms, and suggest that strategies for potentiating the enhancement of endocytosis upon USMB treatment may improve targeted drug delivery.

## Introduction

Conventional drug administration methods such as intravenous injection and oral administration are the main methods for delivering chemotherapeutic molecules to tumor cells. However, poorly organized tumor vasculature, irregular blood flow, high interstitial pressure within the tumor tissue, and broad adverse effects on healthy cells limit the effectiveness of anti-cancer agents [1,2]. An attractive method to enhance the safety and efficiency of drug treatment in cancer is to supplement conventional administration methods with targeted drug delivery strategies in order to enhance drug uptake within tumour tissues while limiting their action within healthy cells [3,4].

A major barrier for the efficacy of many clinically relevant anti-cancer drugs (e.g. gemcitabine, 5-flurouracil, cisplatin) is the passage of these molecules across biological membranes, whether in the context of transit across an endothelial monolayer or across the plasma membrane of the cancer cell itself [5]. Hence, therapeutic strategies that can enhance drug uptake into cells with improved efficiency and specificity are of high importance for drug delivery to treat localized diseases such as cancer.

Ultrasound in combination with microbubbles (USMBs) is a promising strategy for the targeted delivery and enhancement of cellular drug uptake [6–10]. Microbubbles (MBs) consist of a gas core with a diameter of less than 5 μm that is encapsulated by lipids, albumin, or polymer. Ultrasonically-stimulated microbubbles induce sheer stress on the plasma membrane, a phenomenon that can lead to the local uptake of drugs at the targeted tissue [11,12]. The biological effect of ultrasound and microbubble interaction (USMB) occurs as a result of mechanical forces on the plasma membrane, which leads to transient pore formation or disruption of the plasma membrane (sonoporation) [13]. Collectively, these still poorly understood processes lead to an increase in the uptake of materials such as drugs from the extracellular space [7,8].

It was initially thought that USMB-induced pores were the only passageways for the entry of small molecules. However, it was later reported that an enhancement of endocytosis contributes to the uptake of larger molecules following USMB treatment, as evinced by the observation that USMB treatment elicited the uptake of large dextrans (>155 kDa) into punctate structures in an ATP-dependent manner [12]. This suggests that USMB treatment can elicit both uptake of molecules by pore formation as well as by increased fluid-phase endocytosis. Indeed other studies have also reported that USMB treatment increased fluid-phase uptake of FITC-dextran [13] and also enhanced the formation of internalized vesicles [14]. Treatment with chlorpromazine or genistein reduced the fluid-phase uptake of SYTOX Green upon USMB treatment [15]. While chlorpromazine and genistein have been described as inhibitors of clathrin-dependent and caveolae-dependent endocytosis, respectively, these inhibitors have many other effects. Hence, while some studies have shown an increase in fluid-phase endocytosis upon USMB treatment, the underlying mechanisms that elicit enhanced endocytosis after USMB treatment remain to be resolved.

There are several endocytic mechanisms that operate within virtually all human cells. The best understood of these is clathrin-mediated endocytosis (CME), a process responsible for much of the receptor-mediated uptake of extracellular materials [16,17]. CME initiates by the recruitment of the proteins clathrin, the adaptor protein AP-2 and numerous other cytosolic proteins to a 50–100 nm invaginating region of the plasma membrane termed a clathrin-coated pit (CCP). Following a maturation process, CCPs undergo scission from the plasma membrane, resulting in the formation of intracellular vesicles. Recruitment of receptor proteins (termed cargo) to CCPs leads to internalization of these cargo proteins and their associated extracellular ligands from the cell surface. Recent studies have used total internal reflection fluorescence microscopy (TIRF-M) and systematic image analysis involving automated detection and tracking of CCPs to study the regulation of CCP initiation, formation, assembly and scission. These studies have revealed a complex regulation of cargo receptor and CCP dynamics by lipids such as phosphatidic acid [18], phosphatidylinositol-4,5-bisphosphate [19], and phosphatidylinositol-3,4-bisphosphate [20], as well as by proteins including AP-2 [21], dynamin [21,22] and many others [23]. These studies indicate that various discrete stages in the process of assembly and function of CCPs are each subject to regulation by multiple distinct stimuli. How USMB treatment may control CME and the properties of CCPs is not known.

In contrast to the uptake of receptor-bound macromolecules *via* CME, fluid-phase uptake occurs largely through clathrin-independent mechanisms in most cells [24–26]. Clathrin-independent endocytosis likely occurs as a result of several distinct endocytic mechanisms, some of which occur constitutively and others that can be stimulated under some conditions like growth factor stimulation (e.g. micropinocytosis). Clathrin-independent endocytosis in fibroblasts is responsible for three times as much fluid-phase uptake as clathrin-dependent processes [27]. Importantly, clathrin-independent fluid-phase uptake represents a significant uptake process for drug molecules, in particular those that do not effectively interact with cell surface receptors or transporters.

A possible mechanism by which USMB may effect control of endocytosis is through the formation of transient membrane pores which form immediately upon USMB treatment and are resealed in less than 30 seconds, leading to Ca^2+^ entry and stimulation of lysosome exocytosis [28]. This mechanism triggered by USMB treatment may have similar effects as observed in the membrane-resealing pathway reported in cells injured with Streptolysin O (SLO), a bacterial pore-forming toxin. Ca^2+^ influx through SLO pores leads to lysosome exocytosis, part of the repair mechanism leading to membrane wound resealing [29–31]. Membrane injury by SLO triggers an increase in endocytosis to remove the toxins from the plasma membrane. The model proposed by Andrews & col. is that upon membrane wounding by SLO, an increase in intracellular Ca^2+^ triggers lysosome exocytosis, releasing lysosomal acid sphingomyelinase to the extracellular space, where it cleaves sphingomyelin to produce ceramide [29,30,32]. Ceramide contributes to the formation of small ordered domains, leading to membrane curvature in supported membrane bilayers [33]. Furthermore, enzymatically produced ceramide (by sphingomyelinase) in the outer leaflet of giant unilamellar vesicles (GUVs) led to the generation of internal vesicles [34]. As such, increasing the concentration of ceramide on the outer leaflet of the plasma membrane may contribute to membrane invagination and vesicle formation [35]. Indeed, blocking acid sphingomyelinase function using the inhibitor desipramine resulted in impaired plasma membrane repair upon SLO treatment [35].

In contrast, other studies have reported a massive clathrin-independent internalization corresponding to ~50% of the plasma membrane in response to an increase in intracellular Ca^2+^, yet this did not require acid sphingomyelinase or ceramide production [32], but may instead depend on large-scale palmitoylation of cell surface proteins [36,37]. Hence, while several mechanisms have been proposed for how pore-formation and an increase in intracellular [Ca^2+^] can facilitate an increase in endocytosis, whether and how USMB treatment may elicit an increase in fluid-phase endocytosis remains poorly understood.

The effective clinical use of USMB as a therapeutic modality requires additional insight into the biological effects of USMB, in particular, the regulation of endocytic pathways. The aim of this study is to investigate the effect of USMB on the rate of CME and fluid-phase uptake, and to examine the role of lysosome exocytosis and acid sphingomyelinase activity in USMB-mediated CME and fluid-phase uptake through the use of specific chemical inhibitors. A better understanding of the mechanisms that underlie the enhancement of endocytic processes upon USMB treatment may provide effective and rational strategies for the enhanced delivery of therapeutic drugs [7,8].

## Results

To study the effect of USMB on endocytosis, we treated retinal pigment epithelial cells (ARPE-19 cells, RPE henceforth) or MDA-MB-231 breast cancer cells with USMB and measured the impact on the membrane traffic of TfR (to measure clathrin-mediated endocytosis), and horseradish peroxidase or fluorescent dextran uptake (to measure fluid-phase uptake). Fluid-phase endocytosis occurs by the internalization of soluble material from the extracellular milieu by the collective function of several endocytic mechanisms, including those that internalize specific receptors (e.g. clathrin, caveolae) and non-receptor mediated mechanisms (e.g. micropinocytosis). As such, while the fluid-phase uptake markers used in this study (horseradish peroxidase, fluorescent dextran) do not interact with cell-surface receptors, their internalization is mediated by the collective action of a number of internalization mechanisms, although the role of clathrin-mediated endocytosis in fluid-phase uptake is minor [27]. RPE cells are an emerging model to study the regulation of membrane traffic processes, given their ease of culture and their amenability to total internal reflection fluorescence microscopy to study cell surface phenomena.

### Ultrasound Microbubble Treatment Rapidly Enhances Clathrin-Mediated Endocytosis

**Fig 1:**
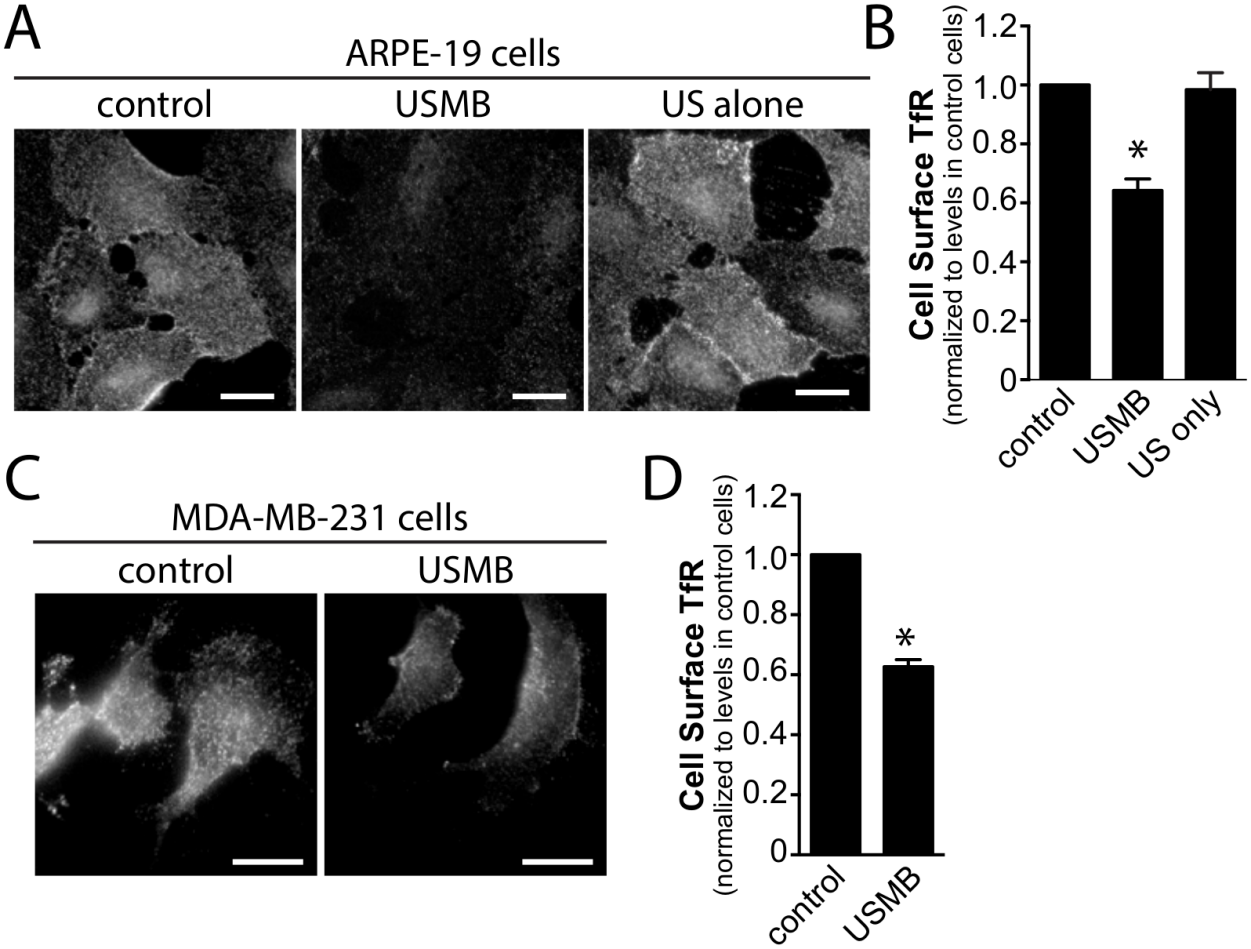
USMB treatment rapidly reduces cell surface TfR levels. RPE (***A*, *B***) or MDA-MB-231 ***(C-D)*** cells grown on glass coverslips were treated with microbubbles and/or ultrasound, as indicated. 5 min following USMB treatment, cells were placed on ice to arrest membrane traffic and subjected to immunofluorescence staining to detect cell surface TfR levels. Shown in ***(A*, *C)*** are representative epifluorescence micrographs of cell surface TfR levels and in ***(B*, *D)*** the mean ± SEM of cell surface TfR fluorescence intensity in each condition (n = 3 independent experiments, each experiment >20 cells per condition). Scale = 20 μm. *, p < 0.05 relative to the control condition.

To investigate whether USMB may regulate the rate of CME, we first examined the cell surface levels of transferrin receptor (TfR), a well-established cargo protein internalized exclusively by CME. We compared the cell surface levels of TfR in control cells to that of cells 5 minutes after USMB treatment. After USMB treatment, the cell surface TfR fluorescence intensity was reduced by 35.3 ± 3.9% compared to cells not exposed to USMB (n = 3, p < 0.05, Fig 1A and 1B). In the presence of US but in the absence of microbubbles, the level of cell surface TfR was indistinguishable from control cells (Fig 1A and 1B), showing that the reduction of cell surface TfR was due to the combined effect of US and microbubbles. Similar results were obtained in MDA-MB-231 cells, in that USMB treatment reduced cell surface TfR levels by 37.28 ± 4.0% compared to control cells not exposed to USMB (Fig 1C and 1D).

**Fig 2:**
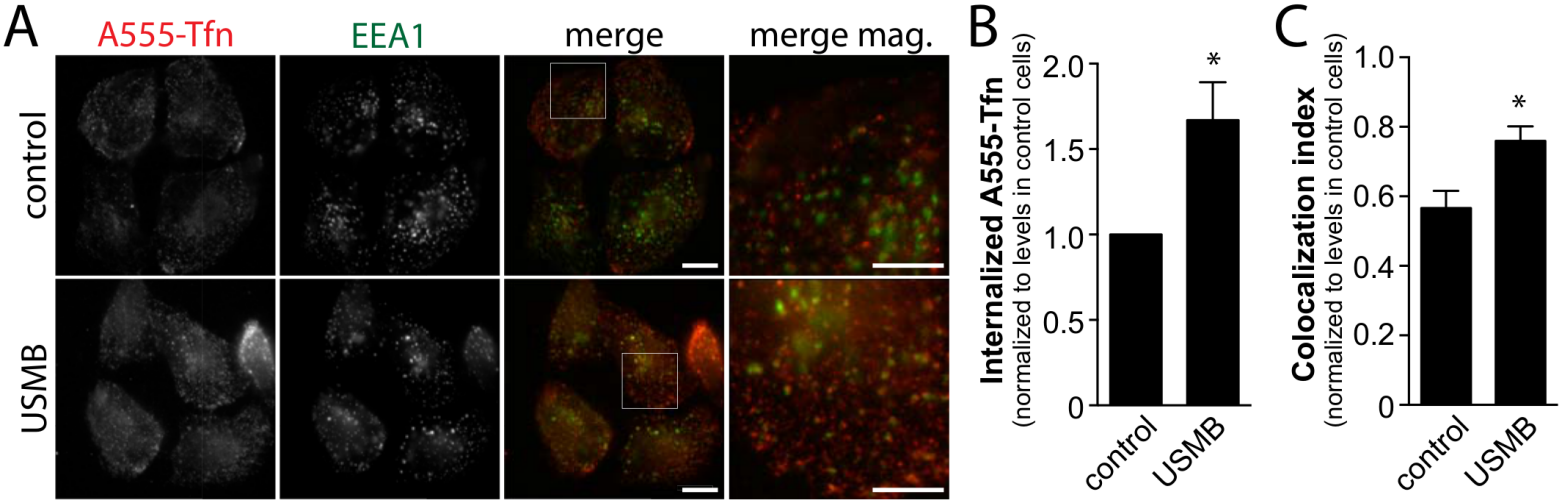
USMB treatment enhances the rate of Transferrin uptake. RPE cells grown on glass coverslips were treated with microbubbles and ultrasound (USMB), or left untreated (control), as indicated. Following treatment, cells were incubated with A555-Tfn for 7.5 min, and then immediately placed on ice, washed and fixed, and subjected to staining to detect EEA1. (***A***) Shown are representative epifluorescence micrographs depicting EEA1 and internalized A555-Tfn. (***B***) Shown are mean ± SEM of internalized A555-Tfn intensity in each condition (***C***) Shown are the mean colocalization index between A555-Tfn and EEA1 (determined by Pearson’s coefficient). For B, C: n = 3 independent experiments, each experiment >20 cells per condition. Scale = 20 μm, Magnified image scale 10 μm. *, p < 0.05 relative to the control condition.

TfR undergoes constitutive CME and recycling [38], such that the reduction in cell surface TfR levels upon USMB treatment could arise from an increased rate of endocytosis or a decreased rate of recycling. To resolve this, we measured the uptake of fluorescent transferrin (Tfn, a ligand of TfR): RPE cells exposed to USMB exhibited an 67.0 ± 22.2% increase in the uptake of Tfn compared to control cells (n = 4, p < 0.05, Fig 2A and 2B). To confirm that the enhanced cell-associated Tfn fluorescence reflected enhanced internalization (and not merely increased binding of A555-Tfn to the cell surface), we quantified co-localization of Tfn with the early endosomal marker EEA1. USMB treatment resulted in an increase in colocalization score between Tfn and EEA1 (Fig 2C). Hence, the increase in fluorescent Tfn labeling in USMB-treated cells during the Tfn uptake assay indeed reflected an increase in Tfn internalization. Moreover, that the extent of co-localization of internalized Tfn with EEA1 increased upon USMB treatment indicates that Tfn uptake was vesicle-mediated and due to not direct cytosolic entry via transient membrane pores, the latter which generally reseal within 30 s after USMB treatment [28]. Hence, the reduction of cell surface TfR upon exposure to USMB resulted from enhanced internalization of TfR, indicating that USMB treatment elicits an enhanced rate of CME.

### Ultrasound Microbubble Treatment Alters the Properties of Clathrin-Coated Pits

**Fig 3:**
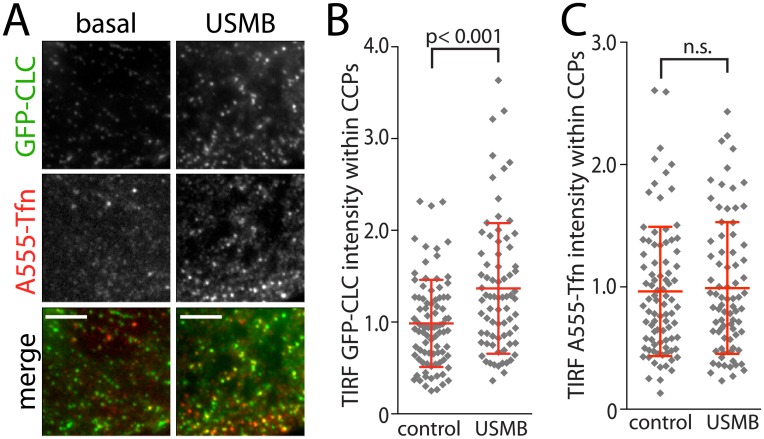
USMB treatment alters the properties of clathrin-coated pits. RPE cells stably expressing clathrin light chain fused to green fluorescent protein (RPE GFP-CLC) cells grown on glass coverslips were treated with microbubbles and ultrasound, or left untreated (control), as indicated. Cells were then incubated with A555-Tfn for 3 min to allow labeling of internalizing TfR, and then immediately subjected to fixation and processing for imaging by total internal reflection fluorescence microscopy (TIRF-M). (***A***) Shown are representative fluorescence micrographs obtained by TIRF-M. Scale = 5 μm. Images are higher magnification insets of larger images shown in S1 Fig. (***B-C***) Images obtained by TIRF-M were subjected to automated detection and analysis of clathrin-coated pits (CCPs), as described in Material and Methods. The mean GFP-CLC (***B***) and A555-Tfn (***C***) intensity within each detected object (CCP) in each cell are shown. Each diamond symbol represents the mean fluorescence of all objects within a single cell; also shown are the mean of the cellular fluorescence values and interquartile range (red bars). The number of CCPs analyzed (n) and cells (k) from 3 independent experiments for each condition are control: n = 37,762, k = 89; USMB n = 29,897 k = 80.

Alterations in the rate of TfR endocytosis could result from changes in the availability of cargo proteins for endocytosis or regulation of CCP formation, assembly, stabilization or scission from the plasma membrane [18,19,21–23,39,40]. To determine how USMB may regulate CCPs, we examined these structures in cells stably expressing clathrin light chain fused to green fluorescent protein (GFP-CLCa); these cells were characterized previously [21] and are henceforth termed RPE GFP-CLC. We performed TIRF-M on RPE GFP-CLC cells that had also been incubated with fluorescently conjugated transferrin (A555-Tfn, to label surface-exposed TfR) for 3 min prior to fixation. Visual examination of TIRF images revealed that USMB treatment appears to increase the fluorescence intensity of clathrin structures (Fig 3A and S1 Fig).

In RPE cells, CCPs are diffraction-limited objects; hence, these structures can be detected using a strategy of estimating the point-spread function using a Gaussian model [21]. This strategy has been previously developed and validated for the systematic detection and analysis of CCPs in fixed images [41] or in time-lapse series [21]. Systematic and automated detection of CCPs in these images revealed that the density of these objects was not changed upon USMB treatment. However, the mean intensity of GFP-CLC within each CCP, determined by the amplitude of the Gaussian model [21,41] was significantly increased in cells treated with USMB compared to control cells (n > 50, p < 0.0001, Fig 3B). The increase in GFP-CLC fluorescence within each CCP indicates an increase in clathrin content per CCP upon USMB treatment, which reflects an increase in the size of CCPs [19]. While the fluorescence intensity of GFP-CLC within each CCP was increased by USMB treatment, that of A555-Tfn (and thus TfR) was indistinguishable between control and USMB-treated cells. The total amount of cell surface TfR was also reduced in USMB-treated cells (Fig 1). Taken together, these data suggests that the efficiency of the recruitment of cargo receptors (e.g. TfR) to CCPs may also be somewhat increased upon USMB treatment or that the proportion of CCPs harbouring TfR that undergo successful endocytosis is increased by USMB stimulation.

These results show that USMB treatment alters the properties of assembly of CCPs at the cell surface, and likely also the efficiency of cargo receptor (TfR) recruitment therein. The vast majority of smaller CCPs are transient structures that undergo abortive turnover without producing an internalized vesicle [21]. Hence, the increase in CCP size upon USMB treatment is consistent with an increased proportion of CCPs leading to the formation of internalized vesicles, and thus an increased rate of TfR endocytosis (Fig 2).

### USMB-Stimulated Enhanced Fluid-Phase Uptake Is Delayed Relative to the Onset of Increased CME

**Fig 4:**
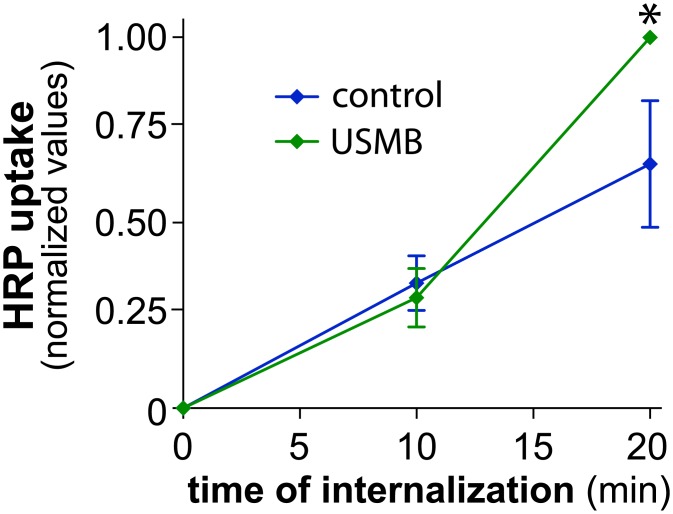
USMB treatment results in a delayed increase in fluid-phase internalization. RPE cells were treated with microbubbles and ultrasound, or left untreated (control), as indicated. Following treatment, HRP uptake was measured as described in *Materials and Methods*. Shown are the mean ± SE of the HRP uptake values at different times following commencement of the assay, which also corresponds to the time following USMB treatment. n = 5, *, p < 0.05 relative to the corresponding timepoint of the control condition.

In order to investigate whether the effect of USMB resulted in specific regulation of CME or whether USMB treatment also controlled other endocytic mechanisms, we studied the rate of fluid-phase uptake after USMB treatment. In most cells, CME has a relatively minor contribution to fluid-phase uptake as other, clathrin-independent mechanisms are largely responsible for this phenomenon [27]. We monitored fluid-phase uptake by measuring the rate of uptake of extracellular, soluble horseradish peroxidase (HRP) [42]. Control cells not treated with USMB exhibited a linear accumulation of intracellular HRP as a function of time. In contrast to the rapid regulation of CME (<5 min) upon USMB treatment, there was no effect on the rate of HRP uptake from 0 to 10 minutes following USMB treatment, compared to control cells (Fig 4). However, HRP uptake was ~2-fold higher in USMB treated cells than control cells at 20 minutes following USMB treatment (n = 5, p < 0.005, Fig 4). These results indicate that in addition to the rapid, nearly immediate increase in the rate of CME, USMB treatment also triggers a relatively delayed increase in fluid-phase uptake. Importantly, that the regulation of these two endocytic pathways (CME and fluid-phase endocytosis) occurs at distinct times following USMB treatment (<5 min and >10 min, respectively) suggests that the mechanisms by which USMB controls each of CME and fluid-phase uptake are distinct.

### Lysosome Exocytosis Occurs after USMB Treatment

**Fig 5:**
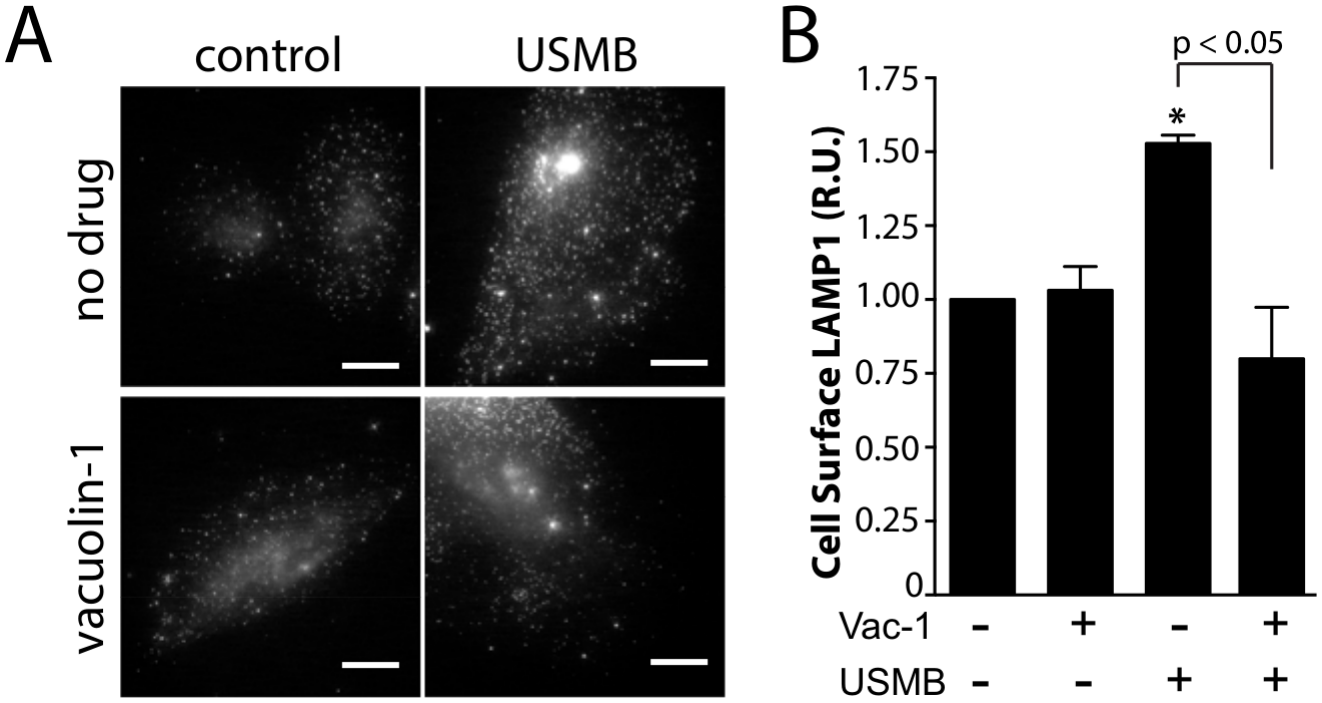
USMB treatment increases the cell surface abundance of the lysosomal marker LAMP1. RPE cells grown on glass coverslips were treated with 5.0 μM vacuolin-1 for 60 min, or not treated with this inhibitor (vehicle control). Cells were subsequently treated with USMB or left untreated (control) as indicated. Following treatment, cells were immediately placed on ice to arrest membrane traffic and subjected to immunofluorescence staining to detect cell surface LAMP1 levels. Shown in ***(A)*** are representative epifluorescence micrographs of cell surface LAMP1 levels and in ***(B)*** the mean ± SEM of cell surface TfR fluorescence intensity in each condition (n = 3 independent experiment, each experiment >20 cells per condition). Scale = 20 μm. *, p < 0.05 relative to the control, vehicle-treated condition.

Lysosome exocytosis occurs upon membrane damage [29] and contributes to the increase in endocytosis upon exposure to SLO [35]. To investigate whether lysosome exocytosis occurs after USMB treatment, we examined the accumulation of the lysosomal marker protein LAMP-1 at the plasma membrane of intact cells. We detected a 1.53 ± 0.16 fold increase in the cell surface level of LAMP-1 in intact cells after USMB treatment, compared to that of control cells (n = 3, p < 0.05, Fig 5A and 5B). This suggests that USMB treatment elicits an increase in lysosomal fusion with the plasma membrane. Treatment with vacuolin-1 (an inhibitor of lysosome exocytosis [43]) completely abolished the increase in cell surface LAMP-1 levels elicited by USMB, as evinced by cell surface LAMP-1 measurements of 1.03 ± 0.05 and 0.80 ± 0.10—fold relative to control in cells treated with vacuolin-1 alone or vaculin-1 and USMB together, respectively (n = 3) (Fig 5A and 5B).

### Vacuolin-1 Inhibits USMB-Stimulated Reduction of Cell Surface TfR but Not the Increase in Fluid-Phase Uptake

**Fig 6:**
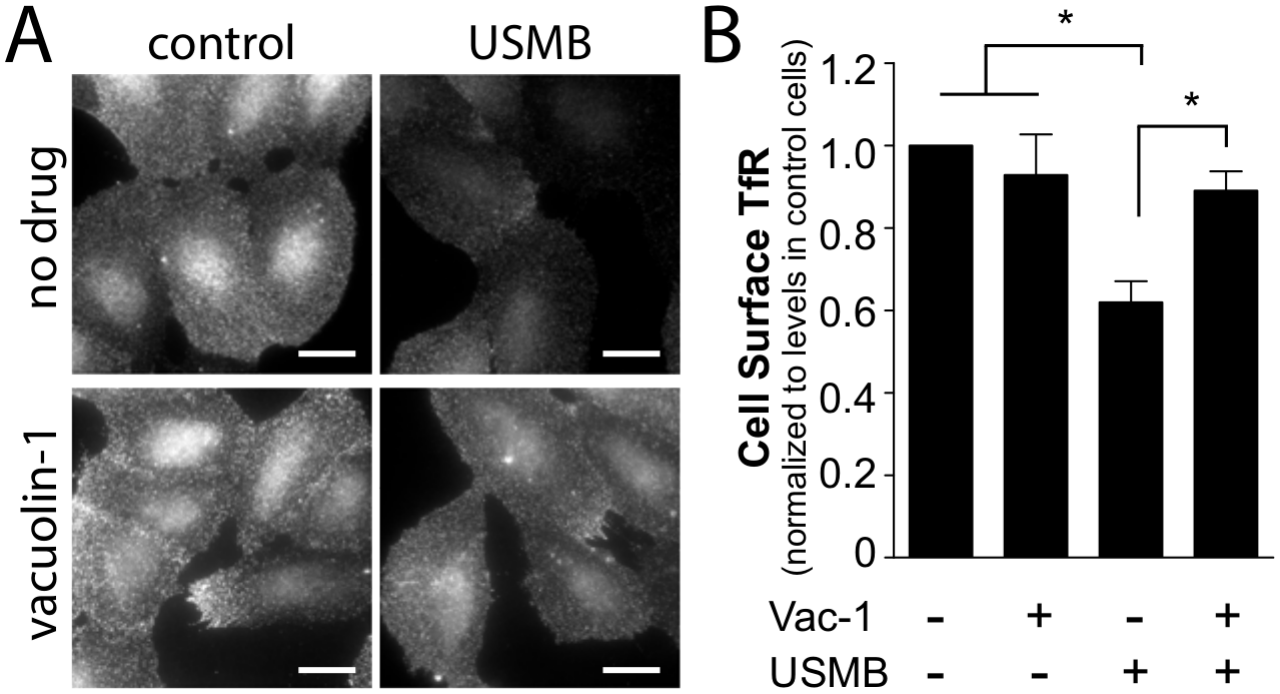
Vacuolin-1 treatment impairs the reduction in cell surface TfR levels by USMB treatment. RPE cells grown on glass coverslips were treated with 5.0 μM vacuolin-1 for 60 min, or not treated with this inhibitor (vehicle control). Cells were subsequently treated with USMB or left untreated (control) as indicated. 5 min after USMB treatment, cells were placed on ice to arrest membrane traffic and subjected to immunofluorescence staining to detect cell surface TfR levels. Shown in ***(A)*** are representative epifluorescence micrographs of cell surface TfR levels and in ***(B)*** the mean ± SEM of cell surface TfR fluorescence intensity in each condition (n = 3 independent experiments, each experiment >20 cells per condition). Scale = 20 μm. *, p < 0.05.

To determine whether lysosome exocytosis contributes to the regulation of CME upon USMB treatment, we examined the effect of vacuolin-1 treatment on the cell surface abundance of TfR upon USMB treatment. While in cells not treated with vacuolin-1, USMB treatment resulted in a 38.0 ± 5.1% reduction in cell surface TfR cells, vacuolin-1 treatment abolished the USMB-elicited reduction in cell surface TfR (n = 3, p < 0.05, Fig 6A and 6B). These results suggest that lysosomal exocytosis may be required for the regulation of TfR by CME upon USMB treatment.

**Fig 7:**
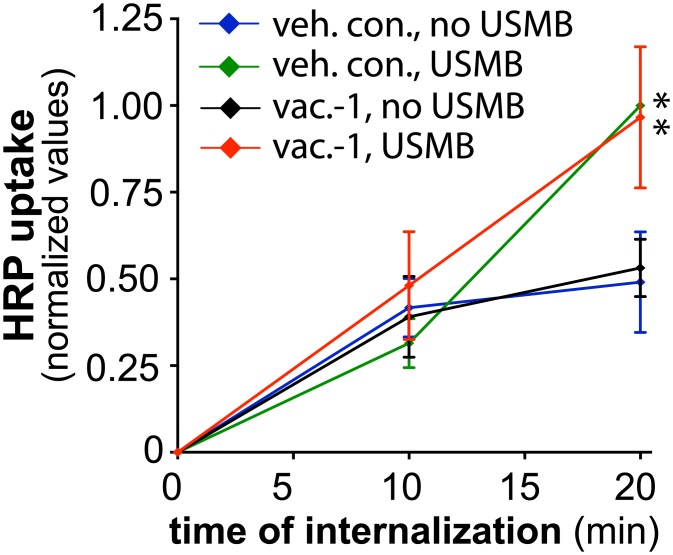
Vacuolin-1 treatment does not affect the regulation of fluid-phase endocytosis by USMB treatment. RPE cells were treated with 5.0 μM vacuolin-1 for 60 min, or not treated with this inhibitor (vehicle control). Cells were subsequently treated with USMB or left untreated (control) as indicated. Following treatment, HRP uptake was measured as described in *Materials and Methods*. Shown are the mean ± SE of the HRP uptake values at different times following commencement of the assay, which also corresponds to the time following USMB treatment. n = 3. *, p < 0.05 relative to the corresponding timepoint of the control condition.

To determine if lysosome exocytosis is also required for the increase in fluid-phase uptake upon USMB treatment, we examined the effect of vacoulin-1 on USMB-stimulated HRP uptake. In contrast to the effect of vacuolin-1 on the USMB-stimulated reduction of cell surface TfR levels, vacuolin-1 treatment had no effect on HRP uptake, either in control cells or cells stimulated with USMB (Fig 7). Collectively, these results indicate that lysosome exocytosis contributes to the regulation of CME but not that of fluid-phase endocytosis upon USMB treatment.

### Desipramine Inhibits the USMB-Stimulated Reduction of Cell Surface TfR

**Fig 8:**
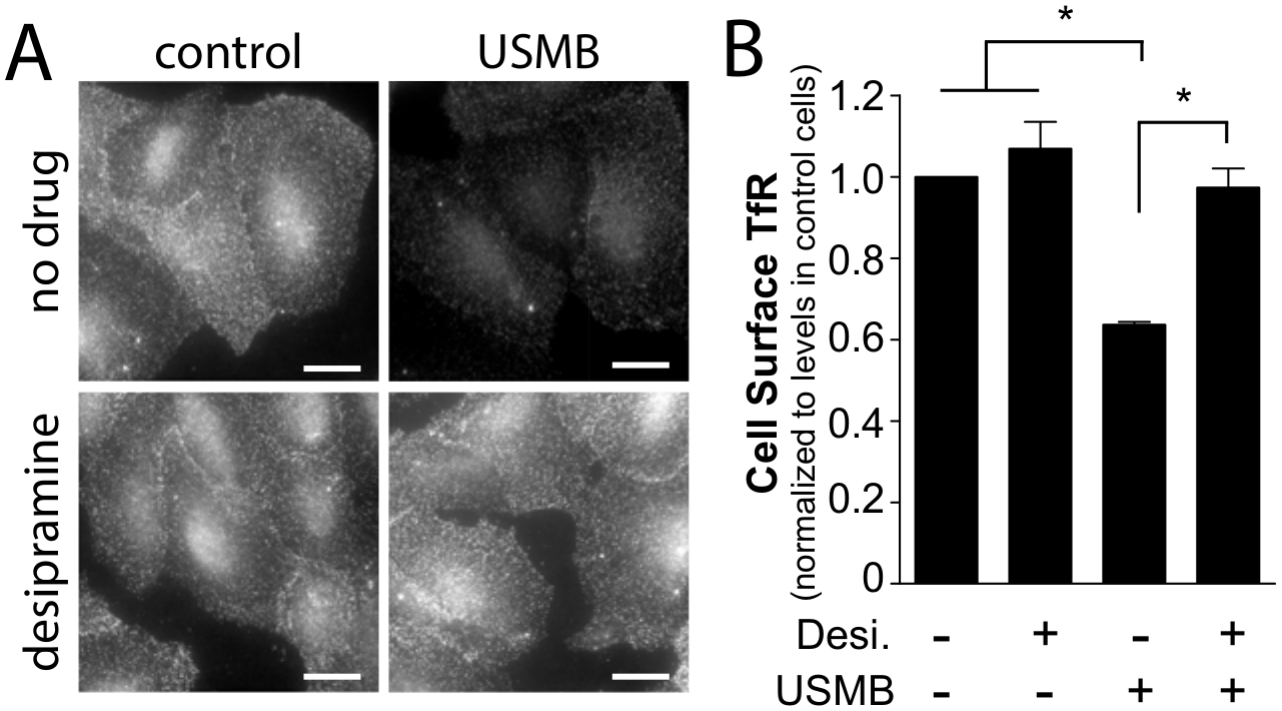
Desipramine treatment impairs the reduction in cell surface TfR levels by USMB treatment. RPE cells grown on glass coverslips were treated with 50 μM desipramine for 60 min, or not treated with this inhibitor (vehicle control). Cells were subsequently treated with USMB or left untreated (control) as indicated. 5 min following USMB treatment, cells were immediately placed on ice to arrest membrane traffic and subjected to immunofluorescence staining to detect cell surface TfR levels. Shown in ***(A)*** are representative epifluorescence micrographs of cell surface TfR levels and in ***(B)*** the mean ± SEM of cell surface TfR fluorescence intensity in each condition (n = 3 independent experiment, each experiment >20 cells per condition). Scale = 20 μm. *, p < 0.05.

In response to membrane damage by SLO, cells undergo membrane repair by enhancing the rate of endocytosis, through the release of acid sphingomyelinase following lysosome exocytosis [35]. As the reduction of cell surface TfR levels upon USMB treatment was sensitive to inhibition of lysosomal exocytosis by vacuolin-1, we aimed to determine if the control of cell surface TfR levels by USMB also requires acid sphingomyelinase. To do so, we treated cells with the acid sphingomyelinase inhibitor desipramine [35]. While control cells (not treated with desipramine) exhibited a 36.3 ± 0.7% reduction in cell surface TfR levels upon USMB treatment (n = 3, p < 0.05), cells treated with desipramine exhibited no detectable change in cell surface TfR levels upon USMB treatment (Fig 8A and 8B). This suggests that the USMB-mediated enhancement of CME of TfR requires acid sphingomyelinase.

### Desipramine Synergizes with USMB Treatment to Enhance Fluid-Phase Uptake

**Fig 9:**
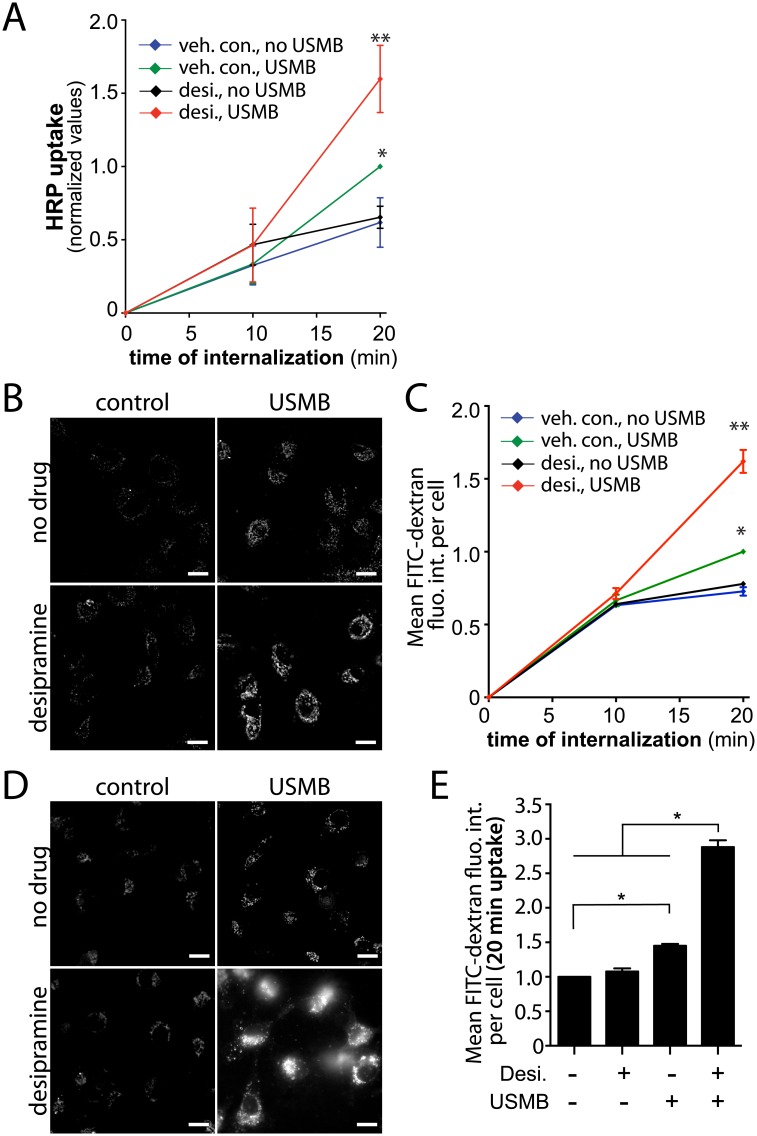
Desipramine enhances the rate of fluid-phase uptake in USMB-treated cells. RPE cells or MDA-MB-231 cells were treated with 50 μM desipramine for 60 min, or not treated with this inhibitor (vehicle control). Cells were subsequently treated with USMB or left untreated (control) as indicated. (***A***) Following treatment, HRP uptake was measured in RPE cells as described in *Materials and Methods*. Shown are the mean ± SE of the HRP uptake values at different times following commencement of assay, which also corresponds to the time following USMB treatment. n = 4, Following treatment, FITC-dextran was measured in RPE cells ***(B-C)*** or MDA-MB-231 cells ***(D-E)*** as described in *Materials and Methods*. Representative fluorescence micrographs of FITC-dextran uptake for 20 min are shown in (B) and (D), scale 20 μm. The mean ± SE of the FITC-dextran values at different times following commencement of the assay, which also corresponds to the time following USMB treatment is shown in (C) and (E) (see S2 Fig). n = 3. * p < 0.01 (relative to vehicle control cells not treated with USMB), ** p < 0.001 (relative to vehicle control cells treated with USMB).

As inhibition of lysosomal exocytosis by vacuolin-1 treatment did not impact the increase in fluid-phase endocytosis upon USMB treatment, we tested whether acid sphingomyelinase activity may also be dispensable for USMB-dependent regulation of fluid-phase endocytosis. Surprisingly, cells treated with desipramine exhibited a robust increase in fluid-phase endocytosis upon USMB treatment compared to cells that were treated with USMB alone (Fig 9A). Similar results were obtained when measuring the effect of desipramine and USMB treatment on the fluid-phase uptake of FITC-dextran (Fig 9B and 9C). Importantly, desipramine treatment alone had no effect on fluid-phase endocytosis (Fig 9A–9C), suggesting that desipramine synergizes with USMB treatment to enhance fluid-phase endocytosis. Notably and consistent with the lack of inhibition of the USMB-stimulated increase in fluid-phase endocytosis by vacuolin-1 treatment, desipramine also failed to inhibit the effect of USMB treatment on fluid-phase endocytosis. We performed similar experiments in MDA-MB-231 breast cancer cells, and found that USMB treatment resulted in a similar increase in FITC-dextran internalization as in RPE cells (Fig 9D and 9E). Also consistent with the results obtained in RPE cells, desipramine alone had no effect on FITC-dextran uptake in MDA-MD-231 cells, but dramatically enhanced uptake of this fluid phase marker in MDA-MB-231 cells also treated with USMB (Fig 9D and 9E). Hence, the regulation of fluid-phase uptake upon exposure to USMB is not restricted to RPE cells, but also occurs in cancer cells such as MDA-MB-231 cells.

These results further support the observation that lysosomal exocytosis is dispensable for the regulation of fluid-phase endocytosis by USMB treatment. Further, this suggests that other cellular targets of desipramine may be attractive targets to improve the ability of USMB to enhance drug delivery by enhancement of fluid-phase endocytosis.

## Discussion

In this study we examined the effect of USMB treatment on the regulation of distinct endocytic processes. By measurement of the cell surface content of TfR (Figs 1, 6 and 8), the cellular uptake of the TfR ligand transferrin (Fig 2), and examination of the properties of clathrin-coated pits using TIRF-M (Fig 3), we found that USMB treatment increased the efficiency of CME and altered the assembly of clathrin-coated pits, resulting in larger structures. Importantly, the changes in these parameters occurred within 5 minutes of USMB treatment, indicating a very rapid regulation of CME upon exposure to USMB.

We measured fluid-phase internalization by monitoring the uptake of soluble HRP followed by enzymatic detection of HRP activity (Figs 4, 7 and 9) or uptake of FITC-dextran and detection by fluorescence microscopy (Fig 9). Using these assays, we found that USMB treatment also increased fluid-phase HRP and FITC-dextran uptake. In contrast to the very rapid, acute regulation of CME upon USMB treatment, we found that fluid-phase uptake was indistinguishable from the control condition for up to 10 min following USMB treatment, while an increase in fluid-phase uptake could be readily observed following 20 min of USMB treatment. This result indicates that the regulation of CME and fluid-phase uptake following USMB treatment occurs by at least partly distinct signaling mechanisms that can be separated based on their response time following USMB treatment.

Using vacuolin-1 and desipramine to inhibit lysosome exocytosis and acid sphingomyelinase, respectively, we found that each inhibited the changes in cell surface TfR upon USMB treatment (Figs 6 and 8). In contrast, vacuolin-1 was without effect on HRP uptake upon USMB treatment (Fig 7), and desipramine enhanced USMB-stimulated HRP and FITC-dextran uptake (Fig 9). These results suggest that while lysosome exocytosis and acid sphingomyelinase may regulate CME upon USMB treatment, these processes are not required for the gain in fluid-phase endocytosis upon USMB treatment. Importantly, these results add to the observation that the regulation of CME and fluid-phase endocytosis by USMB treatment are separated by time, providing further evidence that USMB controls CME and fluid-phase endocytosis by separate mechanisms.

### Lysosome Exocytosis upon USMB Treatment

A seminal study by Andrews & col. showed that membrane wounding by several models, such as during scratch wounding or fibroblast-collagen-matrix contraction assays involves lysosome exocytosis at the site of wounding, measured by staining intact cells with an antibody for the lysosomal protein LAMP1 [29]. Further studies identified that the increase in lysosome exocytosis caused a subsequent increase in endocytosis, which was critical for the repair of wounding lesions such as those triggered by SLO [35]. Consistent with previous studies, we observed that USMB treatment elicited an increase in cell surface LAMP1 staining, indicating increased lysosomal exocytosis. Importantly, cells treated with vacuolin-1, which prevents lysosomal exocytosis [43], did not undergo an increase in cell surface LAMP1 staining upon USMB treatment (Fig 5), further supporting the conclusion that USMB treatment induced an increase in lysosomal exocytosis. Hence, like other treatments that elicit plasma membrane wounding leading to an increase in intracellular Ca^2+^, such as scratch wounding, fibroblast-collagen-matrix contraction wounding, and SLO treatment, USMB treatment elicits an increase in lysosomal exocytosis. This is consistent with a previous study that reported an increase in intracellular Ca^2+^ and an increased cell surface LAMP1 staining upon USMB treatment [28].

### Regulation of CME by USMB Treatment

We found that USMB treatment decreased the levels of TfR at the cell surface by ~35–40%. Given the dynamic nature of TfR membrane traffic, any decrease in the cell surface levels of this receptor might occur as a result of an enhanced rate of TfR endocytosis or a reduced rate of TfR recycling. USMB elicited an increase in the uptake of fluorescent transferrin (Fig 3). Since USMB stimulation elicited both an increase in transferrin internalization and a decrease in cell surface TfR levels, we conclude a direct increase in TfR internalization upon USMB stimulation. That USMB stimulation also increased the colocalization of internalized Tfn with the early endosomal marker EEA1 (Fig 2C) supports this conclusion. Therefore, our results collectively show that USMB treatment increases the rate of TfR internalization, which is known to occur entirely *via* clathrin-mediated endocytosis.

Using TIRF-M, we further uncovered that the increase in TfR internalization correlated with regulation of the properties of CCPs. Our results suggest that USMB treatment alters the assembly or stabilization of CCPs, resulting in an increase in the mean size of CCPs (Fig 3). An increase in the mean CCP size can indicate two non-mutually exclusive effects of USMB on CCPs: (i) an increase in the rate of incorporation of clathrin during the initial assembly phase of CCPs, similar to the effect observed upon increasing the rate of phosphatidylinotiol-4,5-bisphosphate (PIP2) synthesis [19], or (ii) an increase in the proportion of CCPs that are stabilized and become productive for generating vesicles, since these are on average larger than abortive CCPs [21–23,40]. Distinguishing between these possibilities requires measurement of CCP lifetimes and thus time-lapse imaging; however, coupling an ultrasound transducer for near-simultaneous treatment with USMB and imaging by TIRF-M is technically challenging and this instrumentation is not readily available, although some coupled ultrasound fluorescence microscopy systems are being developed [44]. Nonetheless, our results indicate that USMB treatment alters the properties of CCPs in a manner that is consistent with enhanced endocytosis, suggesting that USMB treatment directly controls the formation, assembly or maturation of CCPs.

While USMB stimulation elicited an increase in CCP size, there was no detectable change in A555-Tfn within CCPs upon USMB treatment. We measured a decrease in cell surface TfR levels (Fig 1) and an increase in Tfn internalization (Fig 2) upon USMB stimulation. Taken together, these data indicate that USMB treatment may (i) enhance the recruitment of TfR to CCPs, since in the USMB-stimulated condition, similar levels of TfR are being recruited to CCPs from a smaller pool of cell-surface TfR, and/or (ii) enhance the proportion of CCPs harbouring TfR that form vesicles. Distinguishing between these possibilities again requires measurement of CCP dynamics; however, our results demonstrate that USMB stimulation indeed alters the formation and properties of CCPs and regulates TfR endocytosis.

Clathrin assembly into CCPs, and thus CCP size, is regulated by many factors, including lipids such as PIP2 [19], and clathrin-binding adaptor proteins disabled 2 (dab2) and autosomal recessive hypercholesterolemia (ARH) [39]. Our results indicate that vacuolin-1 and desipramine each prevent the enhancement of TfR endocytosis upon USMB treatment (Figs 6 and 8). This suggests that ceramide produced by acid sphingomyelinase regulates CME, likely by altering the assembly of CCPs. Several studies using a variety of methods have revealed the ability of ceramide to elicit the formation of lipid-ordered domains analogous to lipid rafts [45–47]. Indeed, the addition of sphingomyelinase to lipid bilayers containing sphingomyelin resulted in the formation of lipid ordered microdomains [48]. The formation of ceramide lipid-ordered domains may promote membrane curvature, as ceramide indeed elicits membrane curvature in supported lipid bilayers [33]. Moreover, ceramide synthesis in the outer leaflet of giant unilamellar vesicles produced internal vesicles [34]. Ceramide also promotes transition to non-lamellar membrane phases [49–51], an effect consistent with a role for ceramide in promoting vesicle formation. Efficient CCP assembly and production of intracellular clathrin-coated vesicles requires several proteins that induce and stabilize membrane curvature, such as BAR-domain containing proteins (e.g. amphiphysin, endophilin, SNX9) and Epsin (which contains an ENTH domain). Hence, ceramide produced upon USMB treatment may promote the generation of membrane curvature during CCP assembly or stabilization, although the molecular mechanism by which ceramide may enhance formation of clathrin-coated vesicles remains to be elucidated.

In addition to being an inhibitor of acid sphingomyelinase, desipramine has also been shown to inhibit phosphatidic acid phosphohydrolase, such that treatment with desipramine increases the levels of phosphatidic acid, in turn enhancing the endocytosis of the Epidermal Growth Factor Receptor (EGFR) [52]. However, the regulation of clathrin-mediated endocytosis by phosphatidic acid is selective for that of EGFR but not TfR [18]. Furthermore, desipramine alone was without effect on cell surface TfR levels (Fig 8), which is not consistent with a general enhancement of endocytosis by desipramine treatment. Our results indicate that desipramine treatment reverses the reduction in cell surface TfR levels caused by increased internalization upon USMB treatment, which is consistent with desipramine inhibiting the gain in acid sphingomyelinase activity acting on the outer leaflet of the plasma membrane upon USMB treatment.

Interestingly, in addition to promoting an enhanced uptake of drugs and other molecules found in the extracellular milieu, the increase in endocytosis upon USMB treatment, particular of receptors *via* CME, likely effects broad changes to the protein content of the plasma membrane. Indeed we have previously reported that acute (90 min) activation of AMP-activated protein kinase (AMPK), mimicking conditions that occur during metabolic stress, results in significant changes to the cell surface proteome [53], largely as a result of changes in membrane traffic. USMB is being combined with clinical cancer treatment methods such as radiotherapy and chemotherapy as a means of enhancing treatment effectiveness and safety [9,10,54–56]. Hence, the broad regulation of CME by USMB treatment may also effect large, systematic reprogramming of the cell surface proteome that may have implications for the use of USMB in combination therapy to treat cancer. Thus, it may be important to consider expanding the repertoire of biological effects of USMB to include alterations in the cell surface protein complement of target cancer cells, given the regulation of CME upon USMB treatment.

### Regulation of Fluid-Phase Endocytosis by USMB Treatment

In addition to regulation of CME, we also showed that USMB enhances the rate of fluid-phase uptake. Given that the effect of USMB on the rate of CME can be observed within <5 min after treatment and that an increase in the rate of fluid-phase uptake occurs only > 10 min following USMB treatment, we conclude that USMB differently regulates each endocytic pathway. The increase of fluid-phase endocytosis following USMB treatment did not require lysosome exocytosis, since treatment with the lysosome exocytosis inhibitor vacuolin-1 was without effect on USMB-stimulated HRP uptake (Fig 7). Moreover, the increase in fluid-phase uptake upon USMB treatment was not reversed by treatment with the acid sphingomyelinase inhibitor desipramine, indicating that unlike regulation of CME, the regulation of fluid-phase internalization by USMB treatment did not depend on lysosome exocytosis and acid sphingomyelinase.

How might USMB trigger an increase in fluid-phase internalization? Recent work by Hilgemann & col. indicates that large increases in intracellular [Ca^2+^] elicit massive endocytosis (MEND), and that such a response is independent of CME and can occur independently of lysosome exocytosis and acid sphingomyelinase [32,57]. Recently, mitochondrial permeabilization and membrane protein palmitoylation were shown to contribute to Ca^2+^-induced MEND in some situations [36,37]. Importantly, these responses occurred within ~1 min of a large increase in intracellular Ca^2+^. We observed an increase in fluid-phase internalization only after >10 min following USMB treatment, suggesting that this may involve MEND-like fluid phase internalization with kinetics distinct from previous reports or a distinct mechanism. Another study used electron microscopy and found that USMB stimulation elicited an increase in a number of non-coated pits associated with the plasma membrane [58]. Since formation of these structures was reversed by genistein treatment, these may be caveolae. However, genistein has effects other than inhibition of caveolae. Moreover, mechanical stress (which cells experience upon USMB treatment) triggers disassembly of caveolae without vesicular internalization [59], making it unclear how USMB may increase the number of caveolae at the cell surface and how this could lead to enhanced endocytosis. Hence, while future studies should examine which endocytic mechanism is enhanced by USMB stimulation to effect the increase in fluid-phase endocytosis (e.g. caveolae, micropinocytosis), our results show that USMB treatment elicits an increase in fluid-phase internalization through a mechanism distinct from that used to increase CME, and that this increase in fluid-phase internalization is at least partly distinct from previous reports of increased endocytosis linked to membrane repair.

### Synergistic Enhancement of Fluid Phase Endocytosis by USMB and Desipramine Treatments

We made the surprising discovery that desipramine treatment elicits a synergistic effect with USMB treatment to increase fluid-phase internalization observed >10 min after USMB treatment. In addition to inhibition of acid sphingomyelinase, desipramine can potentiate increases in intracellular [Ca^2+^] elicited by other stimuli [60]. Hence, desipramine may increase the ability of USMB to enhance fluid-phase uptake as a result of enhancing the USMB-stimulated increase in intracellular [Ca^2+^]. Desipramine also causes disruption of cholesterol-rich membrane microdomains, thus impacting the membrane traffic of certain viruses following internalization [61]. It is also possible that the increase in fluid-phase internalization seen upon treatment with USMB reflects delayed induction of micropinocytosis or other actin-dependent mechanisms, although this has not been directly examined.

While the mechanism(s) by which desipramine synergizes with USMB treatment to enhance fluid-phase internalization remain to be elucidated, our findings indicate that specific drug treatments combined with USMB stimulation can greatly enhance fluid-phase internalization. This has important potential implications for applications of USMB for localized enhancement of drug delivery. Specifically, since desipramine did not impact fluid-phase endocytosis on its own but instead only amplified the increase in fluid-phase internalization upon USMB treatment, this suggests that desipramine (or similar compounds) could be administered systemically, followed by highly localized USMB treatment in a tumor to enhance drug delivery. Indeed, we observed a similar regulation of fluid-phase internalization of RPE cells as was as the breast cancer cell line MDA-MB-231, suggesting that this strategy for enhancing drug delivery is not restricted to a single cell type and may be effective for drug delivery to cancer cells in tumors.

In conclusion, we have identified that USMB treatment specifically increases internalization by two distinct mechanisms: clathrin-mediated endocytosis (CME) and fluid-phase endocytosis. We found that the increase in CME was prevented by inhibitors of lysosome exocytosis and acid sphingomyelinase, suggesting that production of ceramide on the plasma membrane may enhance CME upon USMB treatment. Moreover, we propose that combinations of treatments of USMB and specific compounds such as desipramine may be effective to enhance localized drug delivery compared to USMB alone.

## Materials and Methods

### Materials

Peroxidase from horseradish was obtained from Sigma-Aldrich (Oakville, ON). Desipramine was obtained from Sigma-Aldrich (Oakville, ON) and Vacuolin-1 was obtained from Santa Cruz Biotechnology (Santa Cruz, CA). Antibodies used for immunofluorescence microscopy were as follows: anti-TfR from Santa Cruz Biotechnology (Santa Cruz, CA), anti-EEA-1 from Cell Signaling Technology (Danvers, MA), and anti-LAMP-1from Santa Cruz Biotechnology (Santa Cruz, CA). Alexa 555– conjugated Tfn (A555-Tfn) and fluorescein-conjugated dextran 70,000 MW were both from Thermo Fisher Scientific (Rockford, IL). For ultrasound treatment, microbubbles were obtained from Definity mirobubbles (Lantheus Medical Imaging Inc., Saint-Laurent, QC).

### RPE Cell Culture

Human non-immortalized Retinal Pigment Epithelial (ARPE-19) cells were obtained from ATCC (henceforth RPE cells). All RPE cells were maintained in DMEM F12 supplemented with 10% fetal bovine serum (FBS) and 5% streptomycin/penicillin in a humidified incubator at 37°C and 5% CO_2_. For experiments involving total internal reflection fluorescence microscopy to examine clathrin-coated pit (CCP) properties, RPE cells stably expressing clathrin light chain fused to green fluorescent protein (RPE GFP-CLC) were used. Importantly, RPE GFP-CLC cells were thoroughly characterized previously, and shown to exhibit normal, unperturbed endocytosis, making these cells ideal for experiments requiring fluorescent clathrin labeling under minimally perturbing conditions [21].

### Ultrasound Treatment

Using the monolayer model, cells in six-well plates filled with 13 mL media were exposed to USMB at 500 kHz pulse centre frequency (single element flat transducer with 32 mm element diameter focused at 85 mm and a -6dB beam width of 31 mm at the focal point (IL0509GP, Valpey-Fisher Inc., Hopkinton, MA, USA), 570 kPa peak negative pressure (Pneg), 32 μs pulse duration (16 cycles tone burst) at 1 kHz pulse repetition frequency (PRF) corresponding to 3.2% duty cycle, for 60 seconds. These ultrasound stimulation conditions were previously optimized and characterized; of note, there was >80% cell viability upon exposure to USMB stimulation [11,62]. Immediately prior to ultrasound treatment of each sample, Definity mirobubbles (Lantheus Medical Imaging Inc., Saint-Laurent, QC) were added at a concentration of 10 μL/mL. The Definity microbubbles were activated using a Vialmix for 45 seconds. The setup consisted of an arbitrary waveform generator, connected to a power amplifier (AG series Amplifier., T&C power conversion, Inc., NY), which transmitted the electrical signal to the ultrasound transducer. The transducer was submerged in partially degassed deionized water and focused obliquely at the centre of an acoustic window.

### Immunofluorescence Staining

Immunofluorescence detection of cell surface transferrin receptor (TfR) or LAMP1 abundance was previously described [53]. Briefly, following the USMB stimulation protocol, intact cells were blocked for 15 minutes on ice (to arrest membrane traffic) in a solution of PBS+ containing 3% BSA, followed by labeling with a solution containing an antibody to detect an exofacial epitope (of TfR or LAMP1) for 1h at 4°C. Cells were then washed extensively, fixed in a solution of 4% PFA, followed by quenching of the fixative in a 100 mM glycine solution, and detection of surface-bound primary antibodies with the appropriate secondary antibodies. After extensive washing, coverslips were mounted in Dako fluorescent mounting media (Dako, Carpinteria, CA).

### Fluorescent Transferrin Uptake and EEA1 Immunofluorescence Staining

To assess the rate of transferrin ligand (Tfn) uptake, RPE cells were treated with USMB or left untreated (control), and incubated with transferrin-Alexa Fluor 555 Conjugate (A555-Tfn, 10 μg/ml) for 7.5 min and fixed. For the USMB-treated condition, A555-Tfn was added 60 sec following USMB treatment to ensure prior resealing of membrane pores to limit A555-Tfn uptake to clathrin-mediated endocytosis (see S2 Fig). Following this A555-Tfn uptake and fixation regime, immunofluorescence staining (e.g. of total cellular EEA1) was done as previously described [41]. Briefly, cells were subjected to blocking in 3% BSA in PBS for 15 min, followed by labelling with anti-EEA1 and appropriate secondary antibodies. After extensive washing, coverslips were mounted in Dako fluorescent mounting media (Dako, Carpinteria, CA).

### HRP Uptake and FITC-Dextran Fluid-Phase Uptake Measurement

The uptake of horseradish peroxidase (HRP) was measured by incubating RPE cells with 4mg/ml HRP in PBS containing 20mM HEPES and 0.2% BSA (pH 7.4) for different time points (0, 10 and 20 min) at 37°C. A diagram of the timing of HRP addition relative to USMB stimulation is shown in S2 Fig. Following HRP uptake, cells then were detached with a PBS solution containing 0.1% Pronase on ice. Detached cells were washed to remove excess HRP by centrifugation and suspension (all at 4C) and subsequently permeabilized with a PBS solution supplemented with 0.5% Triton-X100. The HRP activity assay was performed in triplicate with o-phenylenediamine dihydrochloride (OPD) as substrate in a 50 mM Na_2_HPO_4_, 27 mM citrate (pH 5) solution containing the following: 10 mg of OPD and 10 μL of 30% H_2_O_2_. Following formation of a colored product, the reaction was terminated by adding 50μl of 3M H_2_SO_4_, followed by measurement of absorbance at 490 nm using an iMark microplate absorbance reader (BioRad, Mississauga, ON).

The uptake of FITC-dextran was measured by incubating RPE or MDA-MB-231 cells with 10 μg/mL FITC-dextran (70000 MW, Thermo Fisher Scientific) for different time points (0, 10 and 20 min) at 37°C. A diagram of the timing of HRP addition relative to USMB stimulation is shown in S2 Fig. Following FITC-dextran uptake, cells were then washed extensively, fixed in a solution of 4% PFA, followed by quenching of the fixative in a 100 mM glycine solution, and coverslips were mounted in Dako fluorescent mounting media (Dako, Carpinteria, CA).

### Epifluorescence Microscopy and Image Analysis

For Figs 1, 2, 5, 6 and 8, immunofluorescence microscopy was performed using a 63x (NA 1.49) oil objective on a Leica DM5000 B epifluorescence microscope using a DFC350FX camera (Leica Microsystems, Wetzlar, Germany). Images were acquired using Adobe Photoshop (San Jose, CA) and all exposure times and image scaling were equal within an experiment.

For Fig 9, immunofluorescence microscopy was performed using a 60x (NA 1.35) objective on an Olympus IX83 epifluorescence microscope using a Hamamatsu ORCA FLASH4.0 C11440-22CU camera. Images were acquired using cellSens software (Olympus) and all exposure times and image scaling were equal within an experiment.

Cell surface TfR (Figs 1, 6 and 8), cell surface LAMP1 (Fig 5), Tfn uptake (Fig 2) and FITC-dextran uptake (Fig 9) in each cell was quantified using ImageJ software (National Institutes of Health, Bethesda, MD) [63], as previously described [53]. Briefly, regions of interest corresponding to entire cells were manually delineated using Image J, followed by determination of mean pixel fluorescence intensity within each region of interest (cell) (see S3 Fig), as per [53]. Images were 16-bit and typical intensity ranges were between 10–30000 units ensuring pixel intensity saturation did not occur. Each condition in each experiment involved measurement of fluorescence intensity of > 20 cells; these single-cell measurements were used to determine the mean cell surface TfR values for each condition in each independent experiment. Measurements of the mean cell surface TfR, cell surface LAMP1, Tfn uptake, and FITC-dextran uptake in each condition and each independent experiment were subject to one-way analysis of variance (ANOVA) with Newman-Keuls post-test, with p < 0.05 as a threshold for significant difference among conditions (except for measurement of cell surface TfR in MDA-MD-231 cells shown in Fig 1D, which was subjected to a student’s t-test with p < 0.05 as a threshold for significant difference among conditions).

In Fig 2, the Colocalization Index (between internalized A555-Tfn and EEA1) was determined by Pearson’s coefficient, measured using the Just Another Colocalization Plugin (JACoP, [64]) in ImageJ. Pearson’s coefficient values were determined for each cell; these single-cell measurements were used to determine the mean Pearson’s coefficient value for each condition in each independent experiment, resulting in Colocalization Index measure for each. Measurements of Colocalization Index for all independent experiments were subjected to a student’s t-test, with p < 0.05 as a threshold for significant difference among conditions.

### Total Internal Reflection Fluorescence Microscopy (TIRF-M) and Image Analysis

TIRF-M imaging was performed using an Olympus IX81 instrument equipped with a 150x (NA 1.45) objective and CellTIRF modules (Olympus Canada Inc., Richmond Hill, ON) using 491 (50 mW) and 561 nm (50 mW) laser illumination and 520/30, 624/50 emission filters. Images were acquired using a C9100-13 EM-CCD camera (Hamamatsu Corporation, Bridgewater, NJ). Diffraction-limited clathrin-coated pits (CCPs) were detected using custom analysis in Matlab (Mathworks Corporation, Natick, MA) as previously described and validated [21,41]. Briefly, CCPs were detected using a Gaussian-based model method to approximate the point-spread function of diffraction-limited CCPs. Fluorescence intensity corresponding to the enrichment of either GFP-clathrin or fluorescently-conjugated Tfn (Alexa555-Tfn) within these detected objects were determined by the amplitude of the Gaussian model for each structure [21,41]. As such, fluorescence intensity measurements of each of these proteins within CCPs represent enrichment relative to the local background fluorescence in the vicinity of the detected object.

## Supporting Information

S1 Fig Full image panels for TIRF-M images shown in Fig 3. To allow visualization of clathrin structures the images shown in Fig 3 are magnified insets of larger images. Shown in this figure are the full images obtained by TIRF-M corresponding to the magnified image insets (shown by the white boxes) shown in Fig 3. Scale = 20 μm.
(PDF)

S2 Fig Diagram depicting the timing of measurements of membrane traffic used in this study. Shown are diagrams of the timing of the experimental manipulations, starting with the USMB stimulation in each case. *Top panel*: For cell-surface TfR level measurement, USMB stimulation is followed by a 5 min incubation, followed by rapid washing and fixation. *Middle panel*: For A555-Tfn uptake experiments (except for TIRF experiments), USMB stimulation is followed by rapid washes, then by incubation in media with A555-Tfn for 7.5 min, followed by immediate fixation. For TIRF experiments in RPE GFP-CLC cells (Fig 3), A555-Tfn is added for only 3 min prior to fixation. *Lower panel*: For fluid-phase uptake measurements, USMB stimulation is followed by a rapid wash, then by incubation with media containing wither HRP or FITC-dextran for 10 or 20 min, followed by immediate assay end or fixation.
(PDF)

S3 Fig Quantification of cellular fluorescence intensity, used for measurement of cell surface TfR and LAMP1, Tfn uptake and FITC-dextran uptake. RPE cells were subjected to detection of cell surface TfR levels (*top panels*, as per Figs 1, 6 and 8) or uptake of A555-Tfn (bottom panels, as per Fig 2). Shown are representative fluorescence micrographs (left images), scale 20 μm. Shown in the right images are overlays of the fluorescence micrographs with manually selected regions of interest (ROI, red dashed lines) corresponding to the entire cell area of all visible cells in each image, as well as a standard ROI corresponding to coverslip background (BG, yellow dashed lines). As described in *Materials and Methods*, cell surface Tfn, LAMP1 or total internalized Tfn or FITC-dextran was measured by quantification of mean pixel intensity within ROIs (corresponding to visible cells) in each image, followed by subtraction of mean pixel intensity of the BG ROI, in order to obtain the net mean pixel intensity for each cell in each image.
(PDF)
