# Supplementary figures and images for: Ultrasound Microbubble Treatment Enhances Clathrin-Mediated Endocytosis and Fluid-Phase Uptake through Distinct Mechanisms
Source: PLoS One. 2016 Jun 8;11(6):e0156754. doi: 10.1371/journal.pone.0156754 (PMC4898768; doi:10.1371/journal.pone.0156754)

S1 Fig

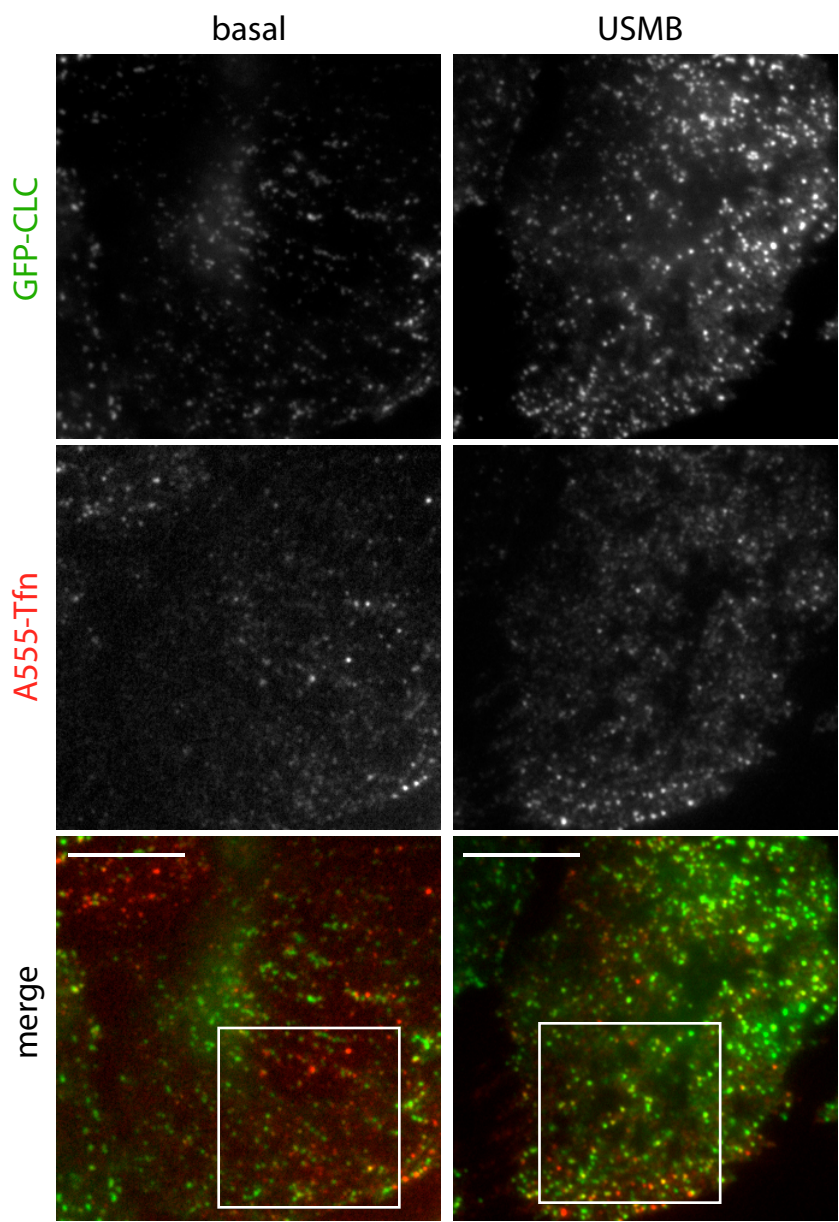

Supplement: S1 Fig — To allow visualization of clathrin structures the images shown in Fig 3 are magnified insets of larger images. Shown in this figure are the full images obtained by TIRF-M corresponding to the magnified image insets (shown by the white boxes) shown in Fig 3. Scale = 20 μm. (PDF) [file pone.0156754.s001.pdf]

S3 Fig

Cell surface TfR quantification in ROIs

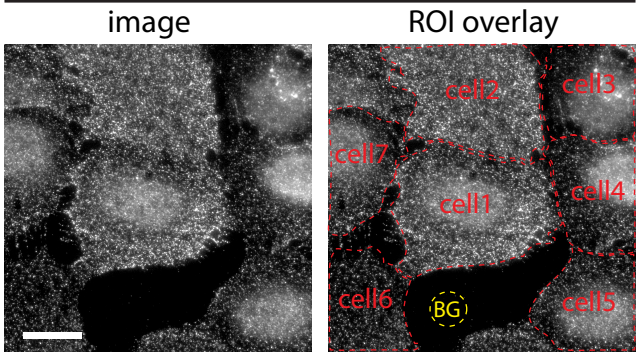

A555-Tfn quantification in ROIs

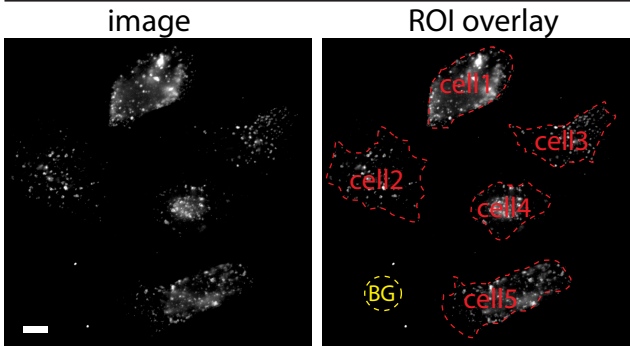

Supplement: S3 Fig — RPE cells were subjected to detection of cell surface TfR levels (top panels, as per Figs 1, 6 and 8) or uptake of A555-Tfn (bottom panels, as per Fig 2). Shown are representative fluorescence micrographs (left images), scale 20 μm. Shown in the right images are overlays of the fluorescence micrographs with manually selected regions of interest (ROI, red dashed lines) corresponding to the entire cell area of all visible cells in each image, as well as a standard ROI corresponding to coverslip background (BG, yellow dashed lines). As described in Materials and Methods, cell surface Tfn, LAMP1 or total internalized Tfn or FITC-dextran was measured by quantification of mean pixel intensity within ROIs (corresponding to visible cells) in each image, followed by subtraction of mean pixel intensity of the BG ROI, in order to obtain the net mean pixel intensity for each cell in each image. (PDF) [file pone.0156754.s003.pdf]
