# Supplementary material for: Ultrasound Microbubble Treatment Enhances Clathrin-Mediated Endocytosis and Fluid-Phase Uptake through Distinct Mechanisms
Source: PLoS One. 2016 Jun 8;11(6):e0156754. doi: 10.1371/journal.pone.0156754 (PMC4898768; doi:10.1371/journal.pone.0156754)

S2 Fig

Cell Surface TfR or LAMP1 measurement (Figures 1, 5, 6, 8)

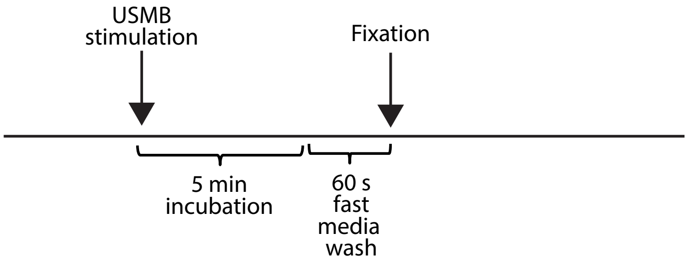

Tfn uptake measurement (Figure 2)

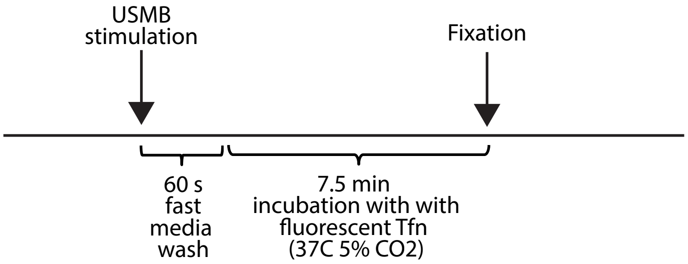

Fluid phase uptake measurements (Figure 4, 7, 9)

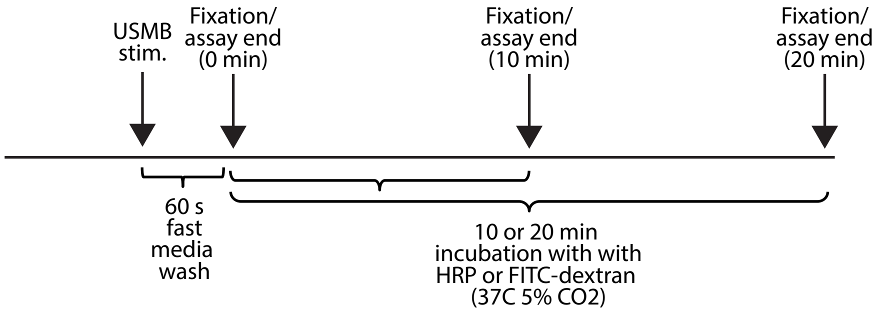

Supplement: S2 Fig — Shown are diagrams of the timing of the experimental manipulations, starting with the USMB stimulation in each case. Top panel: For cell-surface TfR level measurement, USMB stimulation is followed by a 5 min incubation, followed by rapid washing and fixation. Middle panel: For A555-Tfn uptake experiments (except for TIRF experiments), USMB stimulation is followed by rapid washes, then by incubation in media with A555-Tfn for 7.5 min, followed by immediate fixation. For TIRF experiments in RPE GFP-CLC cells (Fig 3), A555-Tfn is added for only 3 min prior to fixation. Lower panel: For fluid-phase uptake measurements, USMB stimulation is followed by a rapid wash, then by incubation with media containing wither HRP or FITC-dextran for 10 or 20 min, followed by immediate assay end or fixation. (PDF) [file pone.0156754.s002.pdf]
